# Childhood adversity and later life prosocial behavior: A qualitative comparative study of Irish older adult survivors

**DOI:** 10.3389/fpsyg.2022.966956

**Published:** 2022-09-07

**Authors:** Shauna L. Rohner, Aileen N. Salas Castillo, Alan Carr, Myriam V. Thoma

**Affiliations:** ^1^Psychopathology and Clinical Intervention, Institute of Psychology, University of Zurich, Zurich, Switzerland; ^2^University Research Priority Program “Dynamics of Healthy Aging”, University of Zurich, Zurich, Switzerland; ^3^School of Psychology, University College Dublin, Dublin, Ireland; ^4^Clanwilliam Institute, Dublin, Ireland

**Keywords:** adverse childhood experiences, resilience, prosocial behavior, altruism born of suffering, qualitative research methods, framework analysis

## Abstract

**Objective:**

Although childhood adversity can have lasting effects into later life, positive adaptations have also been observed, including an increased tendency toward prosocial behavior. However, little is known about the link between childhood adversity and later life prosocial behavior, with a particular scarcity of research on intrafamilial childhood adversity. Therefore, this study aimed to examine older adult's experiences of childhood adversity and identify mechanisms linked to prosocial behavior. Two adversity contexts (intrafamilial and extrafamilial) were compared to explore individual, as well as broader cultural and contextual mechanisms linking childhood adversity and later life prosocial behavior.

**Method:**

Semi-structured interviews (60–120 min) were conducted with *N* = 29 Irish (older) adult survivors of childhood adversity: *n* = 12 intrafamilial survivors (mean age: 58 years, range: 51–72), *n* = 17 institutional survivors (mean age: 61 years, range: 50–77). Interviews were analyzed using the framework analysis method, with reference to the conceptual model of altruism born of suffering.

**Results:**

Five themes were identified on prosocial mechanisms, with three themes in both survivor groups (enhanced empathy, self-identity, amelioration), and two group-specific themes (compassion fatigue in intrafamilial survivors; denouncing detrimental social values in institutional survivors).

**Conclusion:**

Results identified motivational processes and volitional factors linked to later life prosocial behavior. Connections to caring roles, (lack of) support, and social norms in childhood, as well as the need for a sense of purpose and meaning from the adversities in adulthood, highlight potential targets for psychotherapeutic intervention to promote prosocial responding and positive adaptation for childhood adversity survivors.

## Introduction

Childhood adversity refers to potentially traumatic events that occur in childhood and/or adolescence and can be detrimental to health and wellbeing (World Health Organization, 2020a). It is a widespread phenomenon affecting millions of children around the world (World Health Organization, 2020b). Encompassing multiple types of adversity, it can include physical, sexual, or psychological abuse (e.g., Barth et al., 2013; Stoltenborgh et al., 2013), physical or emotional neglect (e.g., Boullier and Blair, 2018; Kobulsky et al., 2020), as well as maltreatment, such as household dysfunction or exposure to violence (e.g., Finkelhor, 2020; World Health Organization, 2020a). Regardless of type, childhood adversity has been systematically associated with lasting negative effects in several domains of health and wellbeing. Physical health effects include neurological disorders, inflammation, and chronic pain (e.g., Huffhines and Jackson, 2019; Petruccelli et al., 2019; Kerr et al., 2021); with mental health effects including personality disorders, depression, and anxiety (e.g., Lindert et al., 2014; Blakemore et al., 2017; Carr et al., 2018). Childhood adversity has also been linked to issues with psychosocial adjustment, such as antisocial behavior, domestic violence, and alcohol and substance abuse (e.g., Fitzpatrick et al., 2010; Afifi et al., 2019; Degli Esposti et al., 2020). However, while adversity type is often the focus of much research, the context in which they occur can also influence how the adversities are experienced (McNeisch and Scott, 2018a,b). For instance, research on intrafamilial (i.e., relatives within the familial environment) and extrafamilial (i.e., non-relatives outside the familial environment) childhood adversity has identified several differentiating factors that can influence the impact of the adversity, including onset, duration, severity, and relationship to perpetrator (Spröber et al., 2014). This complexity highlights the need for research that also considers the adversity environment (Maercker and Horn, 2013). However, studies on extrafamilial childhood adversity often encompass multiple adversity contexts and diverse child-perpetrator relationships (e.g., strangers, neighbors, friends; Magalhães et al., 2009). The current study therefore applies a narrower definition of extrafamilial adversity, focusing on institutional welfare contexts, due to the caregiving definition of the child-perpetrator relationship (analogous to that in intrafamilial contexts).

While early research on childhood adversity had an emphasis on negative outcomes and psychopathology, the focus has expanded to include the potential for positive adaptation and resilience (Bonanno et al., 2011). Resilience has been defined as “the process and outcome of successfully adapting to difficult or challenging life experiences” (American Psychological Association, 2022, para. 1). Prominent resilience researchers have expanded this definition of resilience as a complex construct that may exist on a continuum, be present at differing levels across multiple life domains, and may change over time as a result of the interaction between an individual's development and their environment (Pietrzak and Southwick, 2011; Lehrner and Yehuda, 2018; Ungar, 2018; Masten, 2019). A recently developed theoretical model, the Multi-System Model of Resilience, similarly conceptualized resilience as a capacity that enables functioning on a continuum from vulnerability to resilience, involving the interplay of dynamic coping processes and internal and external resources in response to diverse needs and goals (Liu et al., 2020). Such research emphasizes a strengths- and competence-based approach to overcoming adversity with a focus on positive adaptation rather than psychopathology (Southwick et al., 2014). For example, in an intrafamilial adversity context, a recent qualitative study on resilience by Gunnarsdóttir et al. (2021) interviewed *N* = 22 adult women who had experienced intrafamilial abuse and neglect in childhood. Participants defined themselves as resilient (i.e., experiencing wellbeing and well-functioning) in adulthood, which was described as an ongoing and dynamic process that was supported by personal resources, social supports, establishing command of their lives, and achieving acceptance of their adversity experiences. Similarly, in an extrafamilial institutional adversity context, mixed-methods research by Moore et al. (2019) conducted *N* = 102 quantitative surveys and *N* = 9 follow-up qualitative interviews with Irish adult survivors of clerical institutional childhood abuse. Results identified resilience-enhancing resources associated with mental wellbeing, including problem-focused coping, altruism, social inclusion, and a social identity not defined by institutional care. However, while awareness of the potential for positive adaptation and resilience is increasing, research is still lacking in this area on (older) adult survivors of institutional abuse, and particularly for intrafamilial abuse.

One positive adaptation that is receiving growing research interest is prosocial behavior (e.g., Lay and Hoppmann, 2015; Silke et al., 2018; Martí-Vilar et al., 2019; Pfattheicher et al., 2022), defined as voluntary actions or behaviors intended to help or benefit others (Eisenberg and Miller, 1987). Penner et al. (2005) proposed a multilevel approach to understanding prosocial behavior across three interrelated levels: (1) micro-level intraindividual processes, such as biological mechanisms; (2) meso-level interpersonal processes, such as the relationship between the helper and recipient; and (3) macro-level intra- and intergroup processes, such as volunteering and cooperation. This multilevel perspective suggests that a variety of mechanisms form the foundation for prosocial behavior, including biological and evolutionary processes (e.g., helping relatives, reciprocal altruism for non-relatives), goals and motivational processes (e.g., egoistic and altruistic motives), affective and cognitive processes (e.g., empathy, socialization), and situational, social, and personal processes (e.g., cost-reward calculations, social norms, prosocial traits) (Penner et al., 2005; Dovidio et al., 2006). Interactions within and between these processes are proposed to lead to individual differences in the tendency toward prosocial behavior (Penner, 2002).

As the capacity for prosocial behavior has been shown to emerge early in life (e.g., Eisenberg et al., 2015), it could be expected that disruptions in the caregiving environment, such as childhood adversity, at this developmental stage would impact the development of prosocial behavior. Indeed, some research has indicated a lower probability of exhibiting prosocial behavior for individuals with childhood adversity experiences. For instance, research by Carvalho et al. (2020) examined a community sample of *N* = 673 young adults and found that childhood adversity was linked to the inhibition of altruistic attitudes, an aspect of prosocial behavior. However, other studies have found positive associations between adversity and prosocial behavior, as well as links to resilience. For example, an eight-year longitudinal study examined the role of protective factors in the developmental trajectories of *N* = 449 maltreated children. Results found that despite maltreatment, children with better prosocial skills showed resilient behavioral functioning (i.e., more positive externalizing behavioral adjustments) over time (Yoon, 2018). Furthermore, a qualitative study by Sheridan and Carr (2020) investigated posttraumatic growth after childhood institutional abuse in *N* = 9 adult survivors. Results identified several factors linked to positive change, including the prosocial motivation to help others with similar adversity experiences of social exclusion and marginalization. Mixed-methods research by Frazier et al. (2013) examined the relationship between trauma exposure and prosocial behavior in *N* = 1,528 undergraduate students. Results showed that those who had experienced more lifetime traumas engaged in more helping behaviors and volunteer activities, and that this prosocial behavior was linked to increased wellbeing. Associations with positive affect, perceived meaning in life, and life satisfaction, as well as with PTSD symptoms, were interpreted as helping behavior being motivated by a desire to reduce distress. The qualitative data indicated that negative life events acted as a general motivation for volunteering, as well as evidence of reciprocity motives for prosocial behavior (Frazier et al., 2013). More recent research by Varma and Hu (2022) conducted two experimental lab studies to examine whether prosocial behavior promoted resilience by alleviating trauma-related symptoms following a traumatic exposure. Results found that prosocial behavior (i.e., performing charitable donations) reduced involuntary traumatic intrusions in both lab settings and in daily life, with a mediation analysis suggesting that this was partly due to enhanced positive affect. However, given the limited and conflicting findings on the relationship between childhood adversity, prosocial behavior, and resilience, further research is needed to examine this relationship and explore the underlying processes that may foster an increased tendency toward prosocial behavior.

With childhood adversity linked to both a diminished and enhanced engagement in prosocial behavior, research attention is shifting toward an understanding of the underlying mechanisms that may explain the differing outcomes. One theoretical concept that attempts to describe this relationship between adversity and prosocial behavior is *altruism born of suffering* (Staub and Vollhardt, 2008). It suggests that individuals who have experienced suffering may develop a strong motivation to help others, not only in defiance of the negative experiences, but because of them. Building on this concept, Vollhardt (2009) proposed a model of motivational processes and volitional factors linking suffering and prosocial behavior. Motivational processes strengthen the motivation to engage in prosocial behavior and include: (1) helping as a form of coping or posttraumatic growth (e.g., deflect attention from own problems, find meaning or value in life); (2) helping due to situational demands or social norms; and (3) helping due to positive affect (e.g., relieve distress for self or others) or empathy (e.g., help those with similar adverse experiences) (Vollhardt, 2009). Volitional factors strengthen (or alternatively inhibit) these motivational processes and include aspects such as awareness of injustice, emotion regulation abilities, and (un)supportive environments (Vollhardt and Staub, 2011). Given the inconsistent findings on childhood adversity and prosocial behavior, and the lack of (comparative) research with extrafamilial and intrafamilial survivors; this conceptual framework was applied as a theoretical reference in the analysis of the current study.

The current study therefore aimed to explore the link between childhood adversity and later life prosocial behavior in two groups of Irish older adults. Specifically, it aimed to (a) assess forms of prosocial behavior in survivors of childhood adversity; and (b) identify mechanisms (or motivational processes) underpinning this later life prosocial behavior. This comparative study examined two childhood adversity contexts (intrafamilial adversity and extrafamilial [institutional] adversity) within the same macrosystem, i.e., the society and culture within which the child develops (Bronfenbrenner, 1977). This allowed for an investigation of individual, as well as broader cultural and contextual motivational process linking childhood adversity and later life prosocial behavior in survivors of childhood adversity. As this is a complex subject with limited empirical evidence, a qualitative approach was applied to allow for an open exploration and provide a more comprehensive understanding of the topic.

## Materials and methods

### Study design and procedure

This study was part of a larger cross-sectional, mixed-methods research project on resilience and healthy aging in older adults who experienced childhood adversities. It was conducted in Ireland and led by the University of Zürich, in collaboration with University College Dublin, National College of Ireland, and Ulster University. The current study examined mechanisms associated with prosocial behavior, as identified in the qualitative semi-structured interviews. The study protocol was approved by the Ethics Committee of the Faculty of Arts and Social Sciences in the University of Zürich, Switzerland (ID 18.6.1) and the Human Research Ethics Committee—Humanities in University College Dublin, Ireland (ID HS-18-30-Carr). Informed consent was obtained from all participants in accordance with the Declaration of Helsinki.

### Sample and recruitment

Eligible participants met the following criteria: Irish nationality, native English speaker, 50 years of age or older, with a history of childhood abuse/neglect. An additional criterion for the institutional survivors was having lived in Irish institutional care in childhood and/or adolescence. Recruitment efforts included online advertisements, interviews with the first author on radio programs, word-of-mouth between survivors, and local distribution of flyers in public areas, as well as survivors' groups. Sample size was calculated using empirical research recommendations on theoretical saturation, with a minimum of 12 interviews required to reach theoretical saturation sufficient for emergent themes (Guest et al., 2006). To participate in the study individuals could contact the research team by telephone or email, leading to a screening process for fulfillment of the inclusion criteria. Eligible participants then completed a semi-structured qualitative interview. Two individuals did not meet the inclusion criteria and a further two individuals dropped out prior to interview due to personal reasons. For the interview study, a second screening took place and eligible participants were assigned to one of two groups: survivors of institutional or intrafamilial childhood adversity. During screening, the two groups were matched for adversity using scores on the Adverse Childhood Experience—International Questionnaire (ACE-IQ; World Health Organization, 2020a).

### Data collection

The interview process lasted from August to December 2018 and involved six researchers, including the first author. An interview schedule was developed as a guideline (for further details see Mc Gee et al., 2020), with standardized questions but a flexible order, to ensure all important topics were addressed (Kallio et al., 2016). The interview schedule was pilot tested and refined through three interviews supplemental to the final sample. The resulting interview schedule consisted of an introductory section outlining the interview details and procedure, followed by four content-based topics on: (1) Experiences during childhood and/or adolescence, referring to details of their childhood adversity; (2) dealing with these experiences, referring to coping and support; (3) negative and positive outcomes of these experiences and contributing factors; and (4) reflections on life after the experiences, referring to coping in later life, disclosure of adversity, current stressors, changes in beliefs, and views on healthy aging. Participants were then asked if they would like to add anything not yet covered. A debriefing was conducted at the end of the interview, in which the emotional state of the participants was assessed and a list of psychosocial support options (e.g., counseling services) were provided. Interviews were conducted at different locations, depending on participants' preference and mobility (e.g., at their homes, at the university, *via* Skype). Participants could have an accompanying person present during the interview, which two participants availed of, although this person did not take part in the interview. Interviews were audio-recorded and lasted between 60–120 minutes, with one interviewer conducting the interview and one interviewer taking field notes. At the end of each interview, a small financial compensation (€20) was offered to participants for their time and travel costs. This was not announced during recruitment and was only disclosed to participants after completion of the interview.

### Data analysis: Framework analysis

The interviews were transcribed verbatim using the software f4transkript (Audiotranskription, 2021), and then reviewed and anonymized by two native English (Irish dialect) speakers, including the study lead (first author). Data analysis was conducted using MAXQDA software, version 2020.4.1 was used (VERBI Software, 2018), and following the Framework Analysis method (Ritchie and Spencer, 1994; Gale et al., 2013). This method was employed as it allows for a more systematic and transparent analytical process by following five analysis stages: *familiarization, identification of the thematic framework, indexing, charting*, and *mapping and interpretation* (Ritchie and Spencer, 1994; Srivastava and Thomson, 2009). These five stages result in a highly structured output in the form of a matrix containing the cases, codes, and summaries of the data (Smith and Firth, 2011; Parkinson et al., 2016). See Mc Gee et al. (2020) for a detailed description of these five stages as applied to the qualitative data in this project. Of note for the current study is the theoretical considerations informing the identification of the thematic framework stage, i.e., the motivational process model of altruism born of suffering by Vollhardt and Staub (2011). In framework analysis, a combined theory- and data-driven approach can be used to inform the analysis (Gale et al., 2013).

With regard to the validity and reliability of the findings, at the indexing stage, investigator triangulation was implemented to enhance rigor (Archibald, 2016), with two researchers independently coding all interviews and the final codes and variations being discussed with the study lead (first author). In addition, a selection of transcripts were cross-examined and indexed by two research assistants to strengthen the integrity of the findings. The intercoder agreement was calculated as an indication of the reliability, with a high Kappa value of κ = 0.89 (McHugh, 2012). Finally, to improve the accuracy, credibility, and validity of the findings, member checking (i.e., participant validation; Birt et al., 2016) was conducted with selected participants to discuss and refine the findings and resulting themes.

## Results

In this results section, participant quotes are provided with the following information: participant ID (F = [intra]familial sample; I = institutional sample), gender, and age.

### Sample characteristics

A total of *N* = 29 interviews were conducted with two samples: *n* = 12 survivors of childhood adversity in an intrafamilial context, comprised of 11 females and 1 male aged between 51–72 years (*M*_*age*_ = 57.4 years, *SD* = 6.27); and *n* = 17 survivors of childhood adversity in an institutional context, comprised of 10 females and 7 males aged between 50–77 years (*M*_*age*_ = 60.7 years, *SD* = 7.44). An overview of the socio-demographic information can be seen in Table 1 for the intrafamilial sample and Table 2 for the institutional sample. Regarding the adversities reported by participants, in the intrafamilial sample, emotional abuse (91.7%) and physical abuse (90.9%) were the most reported, followed by sexual abuse (75.0%), emotional neglect (58.3%), and physical neglect (16.7%). In the institutional sample, adversities showed a slightly higher relative frequency of occurrence, with emotional neglect (94.0%) being the most reported, followed by emotional abuse (93.8%), physical abuse (93.3%), physical neglect (93.3%), and sexual abuse (77.8%).

**Table 1:** Participant characteristics for the intrafamilial sample.

**ID**	**Gender**	**Age**	**Marital status**	**Highest level of education**	**Employment status**	**SES**
F01	Female	62	In a relationship	University certificate/diploma	Employed—full time	2
F02	Female	57	Married	University certificate/diploma	Homemaker	5
F03	Female	56	Single	University certificate/diploma	Employed—part-time	–
F04	Female	56	Married	University certificate/diploma	Employed—full time	7
F05	Male	66	Married	Secondary / High school	Retired	6
F06	Female	53	Separated/divorced	University certificate/diploma	Employed—full time	6
F07	Female	72	Widowed	Vocational training	Retired	6
F08	Female	53	Separated/divorced	University certificate/diploma	Unemployed	7
F09	Female	52	Married	University certificate/diploma	Employed—full time	6
F10	Female	51	Married	University certificate/diploma	Employed—full time	5
F11	Female	54	In a relationship	University certificate/diploma	Employed—part-time	7
F12	Female	57	Married	University certificate/diploma	Retired	7

SES, Subjective evaluation of socio-economic status: ranging from 1 (lowest) to 10 (highest). Participant ID uses F to represent the (familial) intrafamilial sample.

**Table 2:** Participant characteristics for the institutional sample.

**ID**	**Gender**	**Age**	**Marital status**	**Highest level of education**	**Employment status**	**SES**	**Years in institutional care**
I01	Female	50	Separated/divorced	University certificate/diploma	Employed—full time	7	14.5
I02	Male	50	In a relationship	Secondary/High school	Homemaker	3	17
I03	Male	77	Married	No formal education	Retired	1	3
I04	Male	51	Separated/divorced	University certificate/diploma	Unable to work	2	5
I05	Male	60	Widowed	University certificate/diploma	Volunteer	5	4
I06	Female	67	Married	University certificate/diploma	Retired	4	<1
I07	Female	66	Separated/divorced	No formal education	Employed—part-time	–	4
I08	Female	61	Single	University certificate/diploma	Volunteer	3	1
I09	Female	63	Separated/divorced	University certificate/diploma	Volunteer	5	7
I10	Female	57	Separated/divorced	University certificate/diploma	Unemployed	2	12
I11	Female	66	Married	Primary school	Retired	5	16
I12	Male	63	Separated/divorced	No formal education	Employed—part-time	3	18
I13	Male	72	Married	Primary school	Retired	7	14.5
I14	Male	53	Single	No formal education	Volunteer	2	12
I15	Female	54	Single	Secondary/High school	Other—carer	2	14
I16	Female	61	Married	University certificate/diploma	Volunteer	1	11
I17	Female	60	Separated/divorced	University certificate/diploma	Employed—full time	3	13

SES, Subjective evaluation of socio-economic status: ranging from 1 (lowest) to 10 (highest). Participant ID uses I to represent the institutional sample.

### Engagement in prosocial behavior

Approximately 83% (10/12) of the intrafamilial sample and 59% (10/17) of the institutional sample reported engaging in later life prosocial behavior, which was identified in three main themes: (1) *Prosocial engagement*, (2) *Volunteering*, and (3) *Social/caring profession*s. The first theme, *Prosocial engagement*, was common in both groups (intrafamilial: 8/12; institutional: 10/17). It referred to actions taken for the benefit of individuals or society, including the general disposition to help others, participation in charitable events, or commitment to a particular cause. For example, stemming from her experiences of “stand[ing] up for the weaker ones” in childhood, one institutional survivor was dedicated to protecting the rights of marginalized groups in later life: “I stand up for all rights, for the elderly, for the health, for everything… say you're, you need something, I'll fight for you.” (I08, female, 61). The second theme, *Volunteering*, was the most systematic prosocial behavior reported, consisting of voluntary, unpaid (or minimally-compensated) activities for the benefit of individuals, a group, or community. More institutional survivors (8/17) engaged in volunteering compared to intrafamilial survivors (3/12). Intrafamilial participants volunteered in different types of organizations (e.g., centers for learning difficulties, youth organizations). In contrast, the majority of institutional participants who volunteered (5/8), did so in survivor-related and survivor-led organizations: “I'd be a really strong staunch activist for the mother and baby homes and Magdalenes.” (I05, male, 60). The second theme, the election of *Social/caring professions* was commonly reported in both groups (intrafamilial: 5/12; institutional: 7/17). Social/caring professions referred to jobs focused on helping or enhancing the wellbeing of others (e.g., nurses, teachers, counselors). As one institutional survivor stated: “Whilst I couldn't be the doctor and nurse that I wanted to aspire to, I still end up in the caring profession, in that I was looking after children… I'm trying to help people to the best of my ability.” (I09, female, 52). In the intrafamilial group, some participants explicitly stated that their adverse childhood influenced their choice of career to help or care for others: “It was hard as a kid growing up, watching my dad like that. So, I think that influenced my decision of a career in later years when I became a nurse.” (F12, female, 57).

### Mechanisms associated with prosocial behavior

The analysis identified five main themes on the mechanisms associated with prosocial behavior. Three themes were identified in both the intrafamilial and institutional survivors: *Enhanced empathy, Self-identity*, and *Amelioration*. In addition, group-specific themes were observed, with *Compassion fatigue* in intrafamilial survivors, and *Denouncing detrimental social values* in institutional survivors. See Table 3 for an overview of the themes.

**Table 3:** Overview of themes—Mechanisms associated with later life prosocial behavior in survivors of adverse childhood experiences.

**Theme**	**Description**	**Example**
**Later life prosocial behavior**		
Prosocial engagement	Tendency toward, interest in, or (informal) actions taken to benefit individuals or society	- Activism, protecting the rights of others - Participation in charitable events - Daily acts of kindness (e.g., buying groceries for a neighbor)
Volunteering	Voluntary, unpaid/minimally-compensated (formal) activities, often structured by an organization	- Voluntary activities in organizations, advice and support centers, survivor-related groups or organizations, etc.
Social/caring professions	Jobs that involve looking after, helping, or enhancing the wellbeing of others	- Nursing, teaching, social work, counseling, etc.
**Mechanisms associated with prosocial behavior**		
Empathy	The ability to recognize or infer what another person is feeling and the corresponding emotional response	- Being able to sense/see pain in others - Perceived uniqueness in the ability to help others as a result of (shared) adverse childhood experiences
Self-identity	Participants' self-perceptions that are shaped by their adverse childhood experiences and linked to their current prosocial attitudes or activities	- Carer identity: Caring for others, being in a caring profession, being strong/resilient and having a responsibility to help those who are weaker - Helper identity: Supporting other's needs, being a people pleaser
Amelioration	Engaging in prosocial behavior to mitigate or lessen the consequences of the adverse childhood experiences	- Having a sense of purpose from engaging in prosocial behavior to help other survivors - Meaning-making: Finding meaning from drawing on their adverse experiences to help others
Compassion fatigue^a^	The feeling of being drained or exhausted from (excessive) prosocial engagement	- Absorbing other people's negative feelings - Being too empathetic
Denouncing detrimental social values^b^	Engaging in prosocial activities that are distanced from / opposed to the detrimental social norms and values of their childhood	- Being a good person (not a religious person) - Advocacy activities, breaking the silence on “taboo” topics in society (e.g., domestic violence)

^a^Theme present only in the intrafamilial sample.

^b^Theme present only in the institutional sample.

#### Enhanced empathy

The theme *Enhanced empathy* was similarly identified in both groups and refers to a heightened ability to recognize or infer what another person is feeling, and the emotional response generated by this perception. The intrafamilial group expressed empathy in the form of an enhanced sensitivity to the suffering of others, which often led to participants acting in a prosocial manner as a source of support for those in need. For instance, one participant reflected on the effects the childhood adversities had on her life, stating that: “I suppose we're all kind of acutely aware of when people are kind of going through things like this, and how they're feeling (…) you can be supportive to somebody, and I think our experiences have kind of made us into people that can be supportive of other people going through that.” (F06, female, 53).

This enhanced empathy and prosocial behavior was also reported in the institutional group, often directed toward other institutional survivors. It was linked to a strong group identification and the belief that they were uniquely suited to helping other institutional survivors: “I mean I know there's some people out there that have never been in institutions and they're great counselors and psychologists. But sometimes, a lot, most of the time, people don't get us.” (I01, female, 50). In both groups, their prosocial attitudes and helping behaviors were facilitated by this increased empathy, which was reported to result from their shared adversity experiences. As stated simply by one participant: “Nobody can do better than the person who's gone through it.” (I09, female, 63).

#### Self-identity

This theme refers to participants' self-perception, which was shaped by their childhood adversity experiences and is linked to their current prosocial attitude or involvement in prosocial activities. Participants identified their childhood adversities as an influencing factor on the development of specific personality traits, which made them more likely to engage in prosocial behavior. For instance, both groups reported having a *carer identity*, defined by caring for others. In the intrafamilial group, the development of a carer identity was attributed to having to take care of or protect (from the perpetrator) other family members, usually siblings or a parent, in the adverse (home) environment of their childhood. This carer self-identity was often reported by participants who later went into social or caring professions: “I work with adults with intellectual disabilities. So, I think the caring for others side of me is there, and that probably has a lot to do with my past, you know, why I do what I do.” (F09, female, 52).

The development of a carer identity in the institutional group was similarly attributed to having to look after other children within the care settings, with one participant stating that “the mammy role kicked you know, being the protector of the smallies” (I01, female, 50). For institutional survivors, their carer identity was also associated with having the strength to survive the adversities and consequences of (institutional) welfare care. This created a sense of responsibility in later life to help to those who were not as strong, through voluntary or outreach work: “If I can protect the vulnerable survivors and try and enable them to be able to say, to help themselves, then that's my overall objective (…) make them realize that they have control of their life now. They're no longer that child back in that place.” (I10, female, 57).

Additionally, a *helper identity* was reported mainly in the intrafamilial group, defined by supporting the needs of others. This was sometimes attributed to the (lack of) support received in their own childhood, leading to an orientation toward supporting other people in later life. For some participants this meant being “much more inclined to be the person that people come to” (F10, female, 51). An extreme case of this helper identity was being a “people pleaser” (F06, female, 53), referring to the disposition to put other people's needs before their own. This was reported by several participants as starting in childhood in an attempt to appease the perpetrator and avoid further abuse or neglect, ultimately becoming an engrained identity trait that transferred into adulthood: “I'm still a people helper. I don't think I can help it. Because it was my coping skill for my father, you would try to please him at all times. (…) If you're in my life, I will try and work my way around your needs before mine.” (F04, female, 56).

#### Amelioration

This theme describes the engagement in prosocial behavior as a means of ameliorating or mitigating their negative childhood experiences. For example, one intrafamilial survivor stated that engaging in charity work later in life “actually made me open up the Pandora's box and deal with my own issues. And it really worked, it was very positive for me.” (F04, female, 56). Both groups described amelioration as involving meaning-making or finding a sense of purpose in the adversities they had experienced. For instance, one institutional participant reflected about his work with a survivor-led organization: “that's keeping me going, I'm trying to do what I can for [survivors]” (I14, male, 53). Participants could draw on their negative childhood experiences and turn it into positive action in later life by engaging in prosocial activities to help others. For example, some intrafamilial participants were involved in a campaign to raise awareness for survivors of intrafamilial abuse and reported that knowing “somebody else is benefitting from your experience of pain” helped them to manage and live with their own adversities (F01, female, 62). Another intrafamilial participant volunteered with children and adolescents, drawing on her experiences to teach about resilience and overcoming adversity: “Yes, I've been treated badly, my childhood has been tough. But do you know what, I'm gonna use this to make sure that other children's childhoods aren't going to be as tough. I'm gonna have them realize yeah, you're in a tough spot. But do you know what? You have the resources within yourself to get above this, and deal with, and use it in your positive.” (F04, female, 56).

#### Compassion fatigue—Intrafamilial survivors

Unique to the intrafamilial group, this theme refers to the feeling of being drained or exhausted from (excessive) prosocial engagement. Often reported by participants in social/caring professions, it was sometimes perceived as a potential inhibitor of further prosocial behavior due to the negative associations elicited. For example, one participant commented that while her childhood adversities made her more aware of when people were going through difficult situations, this also had a negative side: “Sometimes you kind of, you hoover [vacuum] that [negative feelings] up you know, sometimes. And that's, you know, that's not always a great thing either. (…) I just find myself getting very drained by it all (…) you do get very empathetic with people as time goes on. It's not a bad thing, but you do have to be careful of yourself too though.” (F06, female, 53).

#### Denouncing detrimental social values—Institutional survivors

Unique to the institutional group, this theme refers to their need to distance their prosocial attitudes and activities from detrimental social norms and values. For example, when describing their prosocial attitudes in adulthood, participants often made an intentional distinction from the religious values that defined their childhood. This was due to the pervasiveness and power of the church in society and its role in the abuse in the institutions: “Organized religions, I want to know nothing about them. (…) Just trying to do the best I can and not hurt anybody. That's my religion now. It's being kind to everybody, you know.” (I07, female, 66).

Several participants were also involved in prosocial behaviors to oppose the detrimental social norms and values of their childhood, such as advocacy activities to raise awareness about survivor-related causes. Participants felt that by engaging in these activities they defied the culture of silence surrounding institutional abuse, which was fostered by the church, state, and society. For instance, one participant petitioned parliament for an advice and support center for institutional abuse survivors as “the survivor hasn't been seen or heard” (I08, female, 61). Another participant created a short film, drawing on her own experiences, to help and encourage more survivors to speak out: “We did [the film] to inspire and to empower, and break the silence that it's not your fault, you know, it's the society we live in today. But it's up to us to break the silence.” (I16, female, 61).

## Discussion

This study explored the link between childhood adversity and later life prosocial behavior in two survivor groups, focusing on the mechanisms underpinning prosocial behavior. The majority of both the intrafamilial and institutional survivors engaged in prosocial behavior in later life, including general (informal) acts to benefit others, more structured (formal) acts (e.g., volunteering), and the choice of a social/caring profession (e.g., nursing). This is consistent with previous research in trauma populations, including childhood adversity survivors (Hernández-Wolfe, 2011; Bryce et al., 2021; Crann and Barata, 2021). Regarding the mechanisms linked to prosocial behavior, three themes were identified in both groups (i.e., enhanced empathy, self-identity, amelioration), as well as group-specific themes (i.e., compassion fatigue in intrafamilial survivors; denouncing detrimental social values in institutional survivors). Connections were made to their childhood experiences, including the support, caring roles, identity development, and social norms/beliefs. The findings are discussed in detail below, with reference to the conceptual framework (altruism born of suffering; Vollhardt and Staub, 2011), the adversity context, and the broader literature.

Regarding the mechanisms linking child adversity and later life prosocial behavior, the first theme, enhanced empathy, was experienced by participants in both groups as a result of their childhood adversities. This is consistent with previous research linking empathy to prosocial responses intended to benefit others, such as charitable giving or daily acts of kindness (e.g., Vollhardt, 2009; Lim and DeSteno, 2016). For example, Greenberg et al. (2018) conducted two studies in non-clinical samples to investigate childhood adversity experiences and empathy in adulthood. A comparison of adversity and non-adversity groups showed elevated empathy levels in those with childhood adversity, suggesting that these experiences may increase the understanding of others mental and emotional states. This is supported by the current study, as prosocial behavior in intrafamilial survivors was linked to a heightened sensitivity to the suffering of others. While enhanced empathy was also evident in the institutional group, it was rather linked to a strong group identification and survivor-focused prosocial behavior. One explanation may be found in the altruism born of suffering model, which not only identifies increased empathy as a motivator for prosocial behavior, but also the collective experience of suffering and perceived similarities with “ingroup members” (Vollhardt and Staub, 2011). In support of this, another study in this research project with the institutional sample has previously identified “group membership” and “collective identity” as supportive factors for wellbeing (Mc Gee et al., 2020). Therefore, it may be that enhanced empathy, combined with a shared adversity experience and collective group identity (as in the institutional survivors), can be linked to “ingroup” prosocial behavior. While future research is needed examine this further, this finding reinforces the need to consider the implications of the adversity context in the experience of and recovery from childhood adversity.

Regarding the second theme, self-identity, participants reported a carer self-perception, which some linked to their choice of a social/caring profession in later life. This is in line with a recent systematic review on the career choice of helping professionals who experienced childhood adversities (Bryce et al., 2021). Evidence from 28 peer-reviewed studies identified several childhood adversity-related factors associated with a career choice in the helping professions, including family dysfunction, traits developed through the adversity, and parentification (i.e., children assuming the roles and responsibilities of a parent). Consistent with this, participants in the current study reported that their carer identity was shaped in childhood by having to care for other children (i.e., siblings, younger institutional children). Furthermore, a notable finding was the “people-pleaser” helper identity, specific to the intrafamilial sample. This was often attributed to the complexity of the parent-perpetrator relationship and participants' attempts to be agreeable in order to elicit genuine care or affection or simply to avoid further maltreatment. While research on such identity development in intrafamilial abuse is scarce, a recent case study by Edery (2019) examined the influence of narcissistic parenting styles on personality formation and similarly found that being excessively agreeable or a “people-pleaser” started as a childhood coping mechanism. The current findings may therefore fall within the concept of pathological altruism, in which certain extreme prosocial behaviors may represent an unhealthy focus on others to the detriment of the self (McGrath and Oakley, 2012). However, given the lack of research on this topic in intrafamilial adversity contexts, future studies are needed to investigate this further.

The third theme, amelioration, highlighted the importance of prosocial behavior that facilitates meaning-making or generates a sense of purpose from their childhood adversity experiences. This is consistent with the altruism born of suffering model, which proposes regaining meaning as a means of coping with the adversity, which acts as a motivational process for prosocial behavior (Vollhardt and Staub, 2011). Amelioration was also observed in both groups, suggesting that this may be an important coping process for survivors, regardless of adversity context. This is supported by research on meaning-making and sense of purpose through prosocial behavior in survivors of varied adverse experiences, such as intimate partner violence or child sexual abuse (Grossman et al., 2006; Shanthakumari et al., 2014). Therefore, interventions that promote engagement in prosocial behavior may help to provide survivors with an action-oriented means of revising their adversity narrative, by finding purpose in and giving meaning to their experiences (Crann and Barata, 2021).

Unique to the intrafamilial sample, the fourth theme of compassion fatigue described feelings of exhaustion or depletion resulting from (excessive) engagement in prosocial behavior, which could diminish the motivation for further prosocial action. This is comparable to the volitional factor of “emotion control” in the altruism born of suffering model, which suggests that overarousal of emotional states can inhibit prosocial behavior (Vollhardt and Staub, 2011). Furthermore, in the intrafamilial survivors, compassion fatigue was often reported by those in social/caring professions, which is consistent with the main focus of literature on that topic, i.e., with healthcare providers (e.g., Sorenson et al., 2016; Nolte et al., 2017). One explanation for this compassion fatigue in the intrafamilial group may therefore be linked to the potential for burnout, secondary traumatic stress, and vicarious traumatization in such social/caring professions (Sinclair et al., 2017). However, this would not explain why institutional survivors in social/caring professions did not also express compassion fatigue in this study. It may be that compassion fatigue in intrafamilial survivors is exacerbated by the pathological empathy and altruism observed in the extreme and self-sacrificing helper identity (McGrath and Oakley, 2012). However, given that compassion fatigue in survivors of intrafamilial childhood adversity is a novel finding, future research is needed to explore possible connections between the intrafamilial adversity context, personal characteristics (e.g., excessive empathy, emotional regulation), and compassion fatigue.

The fifth theme, denouncing of detrimental social values, was a phenomenon uniquely observed in the institutional group and referred to prosocial attitudes or actions that were in opposition to the defining social norms, beliefs, and practices of their childhood. Given the nature of the abuse in religious-run institutions, this often meant the denouncing of religious teachings in favor of general prosocial values (e.g., being a good person). This is consistent with a study on childhood institutional abuse in Ireland by Sheridan and Carr (2020), in which participants addressed the hypocrisy of the “so-called” Christian society that failed to show kindness or compassion during the abuse. In addition, the current study also found that the majority of institutional survivors engaged in advocacy-related prosocial activities in later life (e.g., awareness-raising, demonstrations, participation in survivor-led organizations, media appearances). In comparison, only a few intrafamilial survivors were involved in such activities (e.g., three participants campaigned for intrafamilial abuse survivors). The connection between the social norms of their childhood (i.e., the culture of silence) and the later life advocacy-related prosocial activities in institutional survivors may be explained by a perceived injustice. In the altruism born of suffering model, a heightened awareness of injustice is proposed to be a volitional factor that reinforces prosocial responses (Vollhardt and Staub, 2011). For example, a study (*N* = 235) by Feather et al. (2012) assessed support for the Stolen Generations, indigenous Australians who were taken from their homes by the state and placed in children's homes or foster care. Results showed that the perception of injustice was associated with increased support for social action. In the current study, although both survivor groups experienced a similar sociocultural context in childhood (i.e., the culture of silence on child maltreatment), in later life institutional survivors received greater social and state recognition of their adversity (e.g., Bergin, 2007; Carr et al., 2010; commission investigation, redress process, media exposure). This systemic and public nature of the institutional adversities may be linked to increased feelings of injustice, and in turn, greater engagement in advocacy-related prosocial activities.

In sum, this study identified motivations underpinning prosocial behavior that emerged from experiences of childhood adversity. This may have important implications for resilience research, as previous studies have shown connections between prosocial behavior and resilient outcomes, including reduced stress, negative affect, psychopathology (e.g., anxiety, depression), and mortality (Haroz et al., 2013; Poulin et al., 2013; Raposa et al., 2016); as well as improved physical and mental health, positive affect, self-esteem, satisfaction with life, and psychological flourishing (Nelson et al., 2016; Moore et al., 2020; Son and Padilla-Walker, 2020; Aydinli-Karakulak et al., 2021). The current findings may highlight the potential role of motivations in the relationship between prosocial behavior and resilience. For instance, regarding advocacy-related prosocial activates, a study on intimate partner violence found that using personal experiences to advocate for other survivors fostered recovery and resiliency through personal healing and empowerment (Flasch et al., 2017). Similarly for meaning-making, research by Banyard et al. (2017) showed that meaning-making was associated with increased health-related quality of life in individuals with high levels of adversity and child abuse. However, engaging in a high number of helping behaviors (i.e., prosocial behavior) was associated with lower health-related quality of life (Banyard et al., 2017), which may be similar to the compassion fatigue observed in the current study. Research on compassion fatigue in oncology nurses found positive associations with empathy, but negative associations with resilience (Cho and Jung, 2014). Although the current study identified some motivations underpinning prosocial behavior, the research depicts a complex picture with resilience. Future studies should aim to build on these findings to further clarify the relationship between adversity, prosocial behavior, and resilience.

The results of this study have some notable implications for policy and practice. Regarding policy considerations, the advocacy and awareness-raising activities of many institutional survivors, such as petitioning parliament, highlight the need for systemic intervention to address this historical issue and improve future institutional care. Meaningful modifications at a governance and institutional level could include public policy reform to create better care programs from within, strengthen the social service workforce and enhance quality of care, as well as develop better control measures to monitor and support minors both during and when transitioning out of institutional care (Newton, 2017). Furthermore, the current study identified “ingroup” prosocial behavior in the institutional survivors, with many involved in survivor-led organizations and some even running their own support groups or survivor networks. The allocation of funding and resources to support such survivor-led measures may promote empowerment and increase the opportunities and capabilities for prosocial responding in these survivors of childhood adversity (Seebohm et al., 2013). Engagement with survivor-led support services was not as evident in the intrafamilial group, which may indicate an aspect of their support system to target with governance-level change and endorsement. This could bring the topic of intrafamilial abuse to public attention, reduce stigma, and encourage survivor-led support in intrafamilial survivors.

Regarding clinical practice implications, the results support the consideration of prosocial activities as part of a trauma-informed approach. Consistent with the current study, research evidence indicates multiple benefits associated with prosocial behavior, such as a sense of purpose, a positive self-concept, feelings of competence, and enhanced wellbeing (Staub and Vollhardt, 2008; Lay and Hoppmann, 2015). Psychotherapeutic programs could incorporate cognitive elements (e.g., facilitating meaning-making of the adversity experience) and behavioral elements (e.g., setting homework tasks of prosocial activities to help others) to develop prosocial behavior and attitudes, strengthen the associated benefits, and promote trauma recovery. Furthermore, interventions tailored to intrafamilial abuse survivors could address the self-detrimental “people-pleaser” identity arising from the complex parent-perpetrator relationship. Interventions such as psychotherapy, psychoeducation, or assertiveness training could help to target this pathological altruism (McGrath and Oakley, 2012).

Some limitations of the current study deserve consideration. First, the study design was retrospective and cross-sectional, which has the potential for recall bias, distortion, or post-event rationalization (Ritchie and Lewis, 2003). However, given the historic nature of the institutional adversity, a prospective study would not have been feasible. Nevertheless, future research could apply different methodological designs, such as longitudinal quantitative assessment, or observational data collection to reduce the risks associated with verbal reports and increase ecological validity (Haynes and Heiby, 2003). Second, both survivor groups are embedded in the Irish cultural context and the results cannot be extrapolated to other populations. Future studies should replicate this study in different cultures, as different norms, values, and socialization practices may lead to differences in prosocial attitudes and responding (Eisenberg et al., 2015). Third, the majority (11/12) of the intrafamilial group was female, which may have influenced some findings. For example, compassion fatigue in social/caring professions may be affected by the female gender bias in some of these occupations (Fielden and Burke, 2014). Further research is needed to examine this in more gender-diverse samples. Fourth, although prosocial behavior was not explicitly mentioned in the recruitment message, the overall study purpose was communicated during recruitment (i.e., a focus on resilience and healthy aging in older adults who experienced childhood adversities). This may have motivated resilient individuals who were already engaging in prosocial activities to participate in the study. In future studies, the careful minimization of potentially influential wording in the recruitment information may facilitate the recruitment of participants with more neutral interests (Sutton and Edlund, 2019). Despite these limitations, some key strengths include the use of a qualitative comparative design, the analytic framing within the conceptual model of altruism born of suffering, and the addition to the limited literature on childhood adversity and prosocial behavior. Research on adaptative outcomes in intrafamilial abuse survivors is particularly scarce, specifically on prosocial behavior, and these findings provide novel considerations for this research topic.

## Conclusion

A total of five main themes were identified on the mechanisms underpinning childhood adversity and later life prosocial behavior in intrafamilial and institutional survivors. Results provide supportive evidence for the motivational processes and volitional factors proposed in the conceptual model of altruism born of suffering (Vollhardt and Staub, 2011). In both groups, enhanced empathy, amelioration, and identity-related mechanisms were linked to prosocial behavior, with connections to the caring roles and (lack of) support in childhood. Aspects of the specific adversity contexts were also linked to prosocial behavior, such as the collective adversity experience for institutional survivors and the parental-perpetrator relationship for intrafamilial survivors. In later life, prosocial behavior in both survivor groups was also driven by the need to turn the negative experiences into positive action, achieved by finding a sense of purpose and making meaning from the adversities. Group-specific mechanisms were also observed in later life, with compassion fatigue hindering prosocial behavior in the intrafamilial group; and the need to oppose the defining detrimental social values of their childhood promoting prosocial attitudes and actions in the institutional group. The shared and group-specific findings highlight the importance of the adversity-context, individual characteristics, as well as socio-cultural influences on the link between childhood adversity and later life prosocial behavior. Further research into these complex mechanisms may help inform the design and development of targeted interventions and promote prosocial responding and positive adaptation for childhood adversity survivors.

## Data availability statement

The original contributions presented in the study are included in the article, further inquiries can be directed to the corresponding author/s.

## Ethics statement

The studies involving human participants were reviewed and approved by the Ethics Committee of the Faculty of Arts and Social Sciences in the University of Zürich, Switzerland (ID 18.6.1) and the Human Research Ethics Committee—Humanities in University College Dublin, Ireland (ID HS-18-30-Carr). The patients/participants provided their written informed consent to participate in this study.

## Author contributions

MVT and SLR were responsible for the conceptualization and methodological design of the project, as well as funding acquisition, project administration, resourcing, and supervising. SLR was responsible for the investigation process and was also involved in data curation and formal analysis together with ANSC. Writing and preparation of the original draft was done by SLR and ANSC, with critical review, commenting, and editing by MVT and AC. All authors read and approved the final manuscript.

## Funding

This work was supported by Swiss National Science Foundation, National Research Program 76, Welfare and Coercion—Past, Present and Future (Grant Number 407640_177355/1), as well as the Swiss Government Excellence Scholarship (ESKAS-Nr. 2016.0109) which funded the first author's (SLR) position.

## Conflict of interest

The authors declare that the research was conducted in the absence of any commercial or financial relationships that could be construed as a potential conflict of interest.

## Publisher's note

All claims expressed in this article are solely those of the authors and do not necessarily represent those of their affiliated organizations, or those of the publisher, the editors and the reviewers. Any product that may be evaluated in this article, or claim that may be made by its manufacturer, is not guaranteed or endorsed by the publisher.
